# Attenuation of Oxidative Stress by Cannabinoids and Cannabis Extracts in Differentiated Neuronal Cells

**DOI:** 10.3390/ph13110328

**Published:** 2020-10-22

**Authors:** Aruna Raja, Soha Ahmadi, Fernanda de Costa, Nan Li, Kagan Kerman

**Affiliations:** 1 Department of Physical and Environmental Sciences, University of Toronto Scarborough, 1265 Military Trail, Toronto, ON M1C 1A4, Canada; aruna.raja@utoronto.ca (A.R.); soha.ahmadi@mail.utoronto.ca (S.A.); 2 Department of Chemistry, University of Toronto, 80 St. George Street, Toronto, ON M5S 3H6, Canada; 3 Lupos Biotechnology Inc., 221 Morrish Road, Toronto, ON M1C 1E9, Canada; fernanda.decosta@lupos.ca (F.d.C.); nan.li@lupos.ca (N.L.)

**Keywords:** antioxidant, SY-SH5Y cells, cannabinoid, THC, CBD, reactive oxygen species, oxidative stress, amyloid-β

## Abstract

In this proof-of-concept study, the antioxidant activity of phytocannabinoids, namely cannabidiol (CBD) and Δ9- tetrahydrocannabinol (THC), were investigated using an in vitro system of differentiated human neuronal SY-SH5Y cells. The oxidative stress was induced by hydrogen peroxide, as reactive oxygen species (ROS). Alzheimer’s disease (AD)-like pathological conditions were mimicked in vitro by treating the differentiated neuronal cells with amyloid-β_1–42_ (Aβ_1–42_) in the presence of Cu(II). We showed that THC had a high potency to combat oxidative stress in both in vitro models, while CBD did not show a remarkable antioxidant activity. The cannabis extracts also exhibited a significant antioxidant activity, which depended on the ratio of the THC and CBD. However, our results did not suggest any antagonist effect of the CBD on the antioxidant activity of THC. The effect of cannabis extracts on the cell viability of differentiated human neuronal SY-SH5Y cells was also investigated, which emphasized the differences between the bioactivity of cannabis extracts due to their composition. Our preliminary results demonstrated that cannabis extracts and phytocannabinoids have a promising potential as antioxidants, which can be further investigated to develop novel pharmaceuticals targeting oxidative stress therapy.

## 1. Introduction

**Figure 1:**
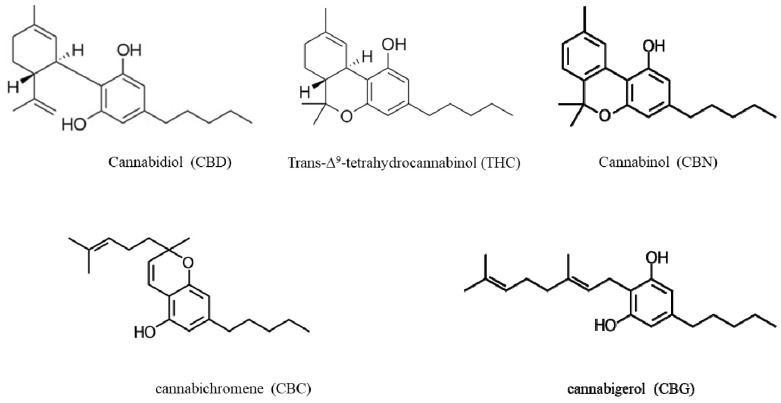
Chemical structures of the main phytocannabinoids found in *Cannabis sativa* L.

One of the oldest known medicinal plants to mankind, *Cannabis sativa L.* (*C. sativa L.*), has been used for various applications spanning over thousands of years [1]. *C. sativa L.* is an annual, dioecious flowering plant. Its first occurrence is believed to be in central Asia [2]. Although the written records of cannabis use both medicinally and recreationally have been found dating back to the 13th Century, extensive interest started in the 19th Century for the preparation of various aliments with anticonvulsive, analgesic, antianxiety and antiemetic properties [3]. Cannabis contains more than 500 natural compounds including oils, proteins, metabolites and fibers [4], of which, more than 200 are metabolites including terpenoids, flavonoids, alkaloids and phytocannabinoids [5]. Phytocannabinoids, a chemical class of C_21_ terpenophenolic compounds produced uniquely by cannabis, are the most bioactive metabolites of cannabis. They have a lipid structure featuring alkylresorcinol, which is classified as neutral cannabinoids (without carboxyl group) and cannabinoid acids (with carboxyl group). Cannabinoids are accumulated as cannabinoid acids and decarboxylated into their neutral forms. Trans-Δ-9-tetrahydrocannabinol (THC; Figure 1) is one of the most potent cannabinoids responsible for the psychoactive effects [6], whereas cannabidiol (CBD, Figure 1) is non-psychoactive [7]. Apart from these, other known phytocannabinoids are cannabinol (CBN), cannabichromene (CBC) and cannabigerol (CBG) (Figure 1), among others [8,9]. Cannabinoids activate a group of receptors in the body called cannabinoid receptors, CB_1_ and CB_2_, which are responsible for various physiological processes, including memory, appetite, mood and pain perception. Prandi et al. [10] provided a comprehensive review of the structure-activity relationship (SAR) mechanisms between cannabinoids and their receptors.

THC is a partial agonist to cannabinoid receptors CB_1_ and CB_2_ [11,12]. The CB_1_ receptor is mainly found in the central nervous system, while CB_2_ is predominant in the immune system [13]. THC is readily absorbed and also passes through the blood-brain barrier due to its lipophilic nature [11]. On the other hand, CBD exerts an anti-inflammatory effect by inhibiting NF-ĸB and interferon-β along with analgesic actions in cancer [14]. CBD has a low binding affinity towards CB_1_ with the capacity to antagonize CB_1_ at nM levels [12]. THC and its analogs have been shown to reduce glutamate toxicity by activating the cannabinoid receptors and reducing the influx of calcium through voltage-sensitive calcium channels [15,16]. We have recently reported on the radical scavenging activity of THC and CBD in the absence of SH-SY5Y cells using UV-Vis assays based on the colorimetric reactions between reactive oxygen species (ROS) and 2,2-diphenyl-1-picryl-hydrazyl-hydrate (DPPH), 2,2-azino-bis(3-ethylbenzothiazoline-6-sulfonic acid (ABTS) and hypochlorous acid (HOCl) [17]. We also applied electrochemical techniques to evaluate the antioxidant activity of THC and CBD using voltammetry [17].

Oxidative stress refers to an imbalance between the production of ROS and the antioxidant defense mechanism causing timely clearance from the biological system [18]. Oxidative stress processes create a significant amount of free radicals. These free radicals are unstable due to the presence of unpaired electrons, which can react freely with biomolecules (lipids, protein, nucleic acid and carbohydrates), causing susceptibility to irreversible damage [19].

Studies performed in vitro have shown that CBD has anti-inflammatory and antioxidant properties [20], as well as reducing tau phosphorylation related to Alzheimer’s disease (AD) [21] and improving cell viability [22]. The ratio of administered CBD to THC significantly influenced these possible effects [23,24]. Different ratios of THC and CBD were utilized in assays such as the estimation the GABA (gamma-aminobutyric acid, a neurotransmitter) levels, Nissl-stained neurons and the mRNA level of antioxidant enzymes such as superoxide dismutase (SOD-1) to judge the neuroprotective effects on the neurons [23,24]. The changes introduced in the expression levels of SOD-1 by 3-nitropropionic acid (3-NP) were completely reversed by using THC and CBD [23,24]. The commercially available form of THC, Dronabinol™, has been used successfully to increase appetite in the case of AIDS and as an antiemetic in the case of cancer chemotherapy [25]. Baker and Pryce [26] recently reviewed the antioxidative and anti-inflammatory properties of CBD. THC has shown neuroprotective effects in animal models of AD, Parkinson’s disease (PD) and amyotrophic lateral sclerosis (ALS) through modulation in glutamatergic transmissions and synaptic plasticity, as well as the modulation of immune response and the excitability of N-methyl-D-aspartate receptors [27].

The key role of cannabinoid receptors in AD makes cannabinoids and cannabis extracts a potential therapeutic candidate for AD [28,29,30]. AD is a progressive degenerative disease of the brain. It is characterized by memory decline, disruption of cognitive ability and eventual loss of bodily function control [31]. Our current understanding of the pathological mechanism of the disease includes aspects such as the involvement of amyloid-beta (Aβ) plaques and the formation of neurofibrillary tangles, along with oxidative stress and inflammatory events, eventually leading to neuronal injury and synaptic loss [32]. For more than two decades, the Aβ hypothesis has been the primary pathophysiology behind AD [33]. According to the amyloid hypothesis, an imbalance between the generation and clearance of Aβ from the brain is the major contributor to the progression of AD pathogenesis [34]. Aβ leads to neuronal lipid peroxidation, protein oxidation and DNA oxidation [35]. Lyras et al. [36] tested different regions of the human brain to assess the level of protein and DNA oxidation. Their results were conclusive concerning the role of oxidative stress in key regions of the human brain implicated in AD. Among other leading causes of AD, metal ion imbalance plays a major role. At the synaptic clefts of AD brains, Aβ_1–42_, Cu(II) (400 mM) and Fe(III) (1 mM) were detected at anomalous levels, causing Aβ aggregation and ROS production [37,38]. Protease resistance, reversible precipitation and the O_2_-dependent production of H_2_O_2_ and concomitant toxicity are all mediated by the affinity of Cu(II) and Zn(II) towards Aβ [39]. Studies have shown that Aβ peptides cause cellular toxicity by generating H_2_O_2_ and O_2_^−^ radicals in vitro [40,41]. Post-mortem AD brain tissue has shown plaques enriched with Aβ_1–42_ in combination with Cu/Zn, and these Aβ-metal complexes were solubilized with the selected metal chelators [42,43]. Furthermore, the interaction of Cu(II) with tau protein also lead to ROS formation and neuronal death [44,45].

Since AD is a multifactorial disease, the therapeutic approach should be targeted at various levels. Most of the experimental evidence as discussed above is available from both cellular and animal models with regard to the potential of cannabinoids. One of the clinical results has shown that treatment with THC analogs resulted in decreased severity in the cognitive behavior of AD patients [46] and increased their appetite [46,47]. Another cellular studies showed that cannabinoids lowered the Aβ levels, inhibited their aggregation and increased mitochondrial function [48,49].

In this study, we investigated the effect of the cannabis extracts, as well as THC and CBD, two main bioactive phytocannabinoids, to combat oxidative stress, one of the hallmarks of AD, using an in vitro model of the neurons. We evaluated the effect of cannabis extracts on the cell viability of the differentiated SH-SY5Y neuronal cell line using fluorescence-based techniques. Our aim in this study was to lay a background for future research with regard to the potential applications of cannabinoids and their synthetic analogs as a novel therapeutic approach to oxidative stress in AD.

## 2. Results and Discussion

### 2.1. Antioxidant Activity

To investigate the antioxidant activity of the phytocannabinoids and cannabis extracts, we developed an in vitro model of AD-like oxidative stress using the differentiated SH-SY5Y cells. We followed two approaches to mimic the AD-like oxidative stress by treating the differentiated SH-SY5Y cells with H_2_O_2_ and also with Aβ_1–42_ and Cu(II) to provide an in vitro model of AD-like oxidative stress.

The in vitro model of AD-like oxidative stress was developed by treating the differentiated SH-SY5Y neuronal cells with two known oxidative stress inducers, namely hydrogen peroxide (H_2_O_2_) [50,51,52] and Aβ_1–42_ in the presence of Cu(II) (Aβ_1–42_-Cu(II) complex) [53,54]. To determine the level of ROS produced by the inducers, DCFDA, a cell-permeable reagent, was used. This reagent was deacetylated by the cellular esterase to an intermediate compound, which was later oxidized by reactive oxygen species (ROS) to 2′,7′-dichlorofluorescein (DCF). This highly fluorescent compound (DCF) was then measured spectrophotometrically at an excitation of 495 nm and an emission of 529 nm.

The effect of H_2_O_2_ is largely mediated by hydroxyl radicals (OH^•^) generated by the Fenton reaction, which is catalyzed by Fe^2+^ [55]. Previous studies have shown that Cu(II) also has an important role in the Fenton reaction to generate ROS [44,45,56]. Postmitotic brain cells such as glial and neuronal cells are particularly sensitive to these free radicals, leading to brain damage [57]. Differentiated SH-SY5Y cells provided us with an ideal model to test the effectiveness of cannabinoids against the oxidative stress environment in pathological conditions. Our previously reported UV-Vis studies showed that THC and CBD had a similar potency using the DPPH, ABTS and HOCl radical scavenging assays [17]. However, the result of this study indicated that THC (IC_50_ = 0.4 µg mL^−1^) had a higher potency in combating the ROS induced by H_2_O_2_ in the SY-SH5Y cells compared to CBD with an IC_50_ of 42.7 µg mL^−1^ (Table 1).

**Figure 2:**
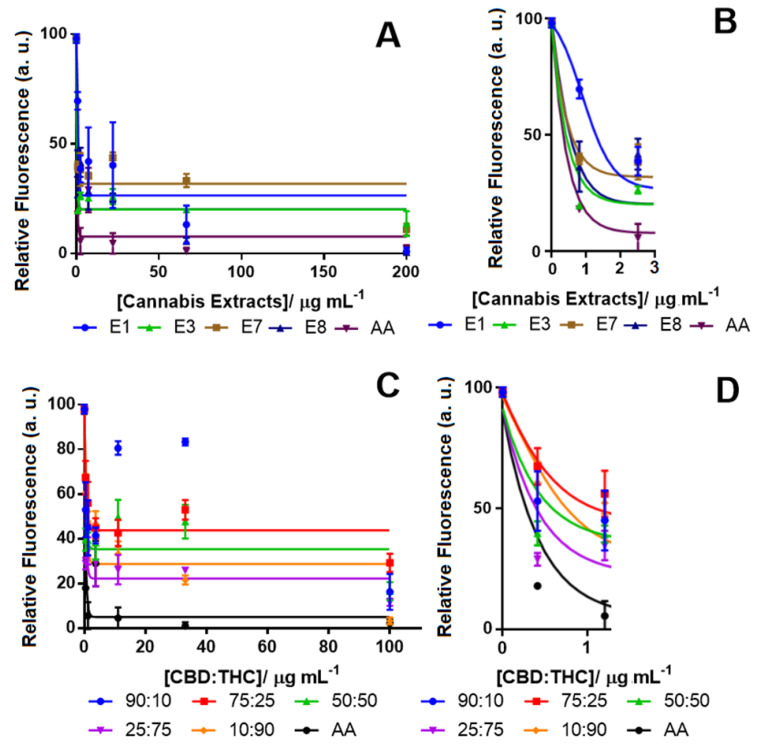
Concentration-response curve for monitoring the ROS level in differentiated SH-SY5Y cells using the 2′,7′-dichlorodihydrofluorescein diacetate (DCFDA) assay after treatment (**A**) with cannabis extracts (tested concentrations for the extracts: 0.0, 0.8, 2.5, 7.4, 22.2, 66.7 and 200.0 µg mL^−1^) and (**B**) the inset; and CBD:THC (**C**) solutions with different ratios of CBD and THC (tested concentrations for the CBD:THC ratios: 0.0, 0.4, 1.2, 3.7, 11.0, 33.0 and 100.0 µg mL^−1^) and along with (**D**) the inset. In all experiments, H_2_O_2_ was used as the ROS inducer (100% fluorescence intensity), and ascorbic acid (AA) was tested in vehicle only (DMSO) as the positive control. Data show the average of the mean values determined in triplicate measurements (*n* = 3). Control (from vehicle only, DMSO) vs. response data (from cannabinoids) are shown using the Bonferroni test at *p* < 0.0001.

The cannabis extracts were also tested using the DCFDA assay to detect their ability to decrease ROS levels after H_2_O_2_ treatment for 24 h (Figure 2A). Table 1 shows the IC_50_ of all the tested extracts and cannabinoid compounds. Ascorbic acid (AA) was applied as a positive control with a known strong antioxidant effect [58,59]. Among all the cannabis extracts, E3 with 71.08% THC and no detectable CBD showed the highest antioxidant activity by reducing the ROS level by 80% (Figure 2A). The other cannabis extracts (E1) that also contained a close amount of THC (72.88%) and no detectable CBD reduced the ROS by more than 70%. Notably, these two extracts also showed similar neurotoxicity (Table 2). Interestingly, extracts E7 and E8, which were poor in THC (11.5% and 3.9%, respectively), were also effective at reducing the ROS by more than 60%. We also examined the effectiveness of the CBD:THC compounds at different proportions (Figure 2B). As expected, the sample mixture with the lowest amount of THC (90:10 of CBD:THC) was less effective at decreasing the ROS level. Our results showed that pure THC (98% pure) reduced the ROS at the same level of AA with an IC_50_ of 0.4 μg mL^−1^, while pure CBD only reduced the ROS by 50% with an IC_50_ of 42.7 μg mL^−1^ (Table 1). These results suggest that other metabolites in the cannabis extracts might be associated with antioxidant activity.

Other constituents of the tested extracts. i.e., CBN, CBG, etc. could either enhance the effects of THC or be reduced by CBD in their antioxidant properties [7]. Studies have shown that in the mixed proportion of CBD and THC, sometimes, CBD hindered the effect of THC, and in other cases, it could potentiate its effect, making it difficult to establish a fixed ratio with the desired potency [24]. Our results also showed that the ratio of CBD:THC at 25:75 showed a high potency with an IC_50_ of 2.5 μg mL^−1^ and was found to be more potent than a CBD:THC ratio of 10:90 with an IC_50_ of 5 μg mL^−1^.

**Figure 3:**
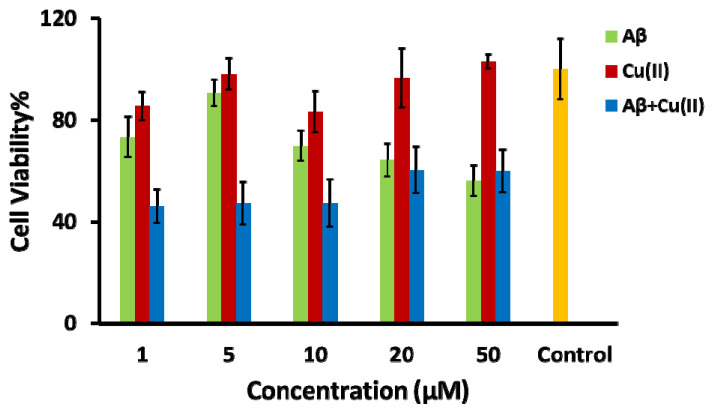
Bar chart illustrating the cell viability, using the MTT assay, of the differentiated SH-SY5Y neuronal cells after 24 h of incubation with amyloid-β (Aβ) (green bar), Cu(II) (red bar), the Aβ + Cu(II) complex (molar ratio 1:1) (blue bar) and control experiments with no treatment of oxidative stress inducers (yellow bar).

It has been shown by several researchers that the interaction of Cu(II) with peptides and proteins leads to ROS formation [44,45,56,60,61]. Therefore, the Aβ-Cu(II) system can be used as an oxidative stress inducer in the SH-SY5Y in vitro model to mimic the AD-like oxidative stress condition [62,63]. Among the Aβ isoforms, Aβ_1–42_ is more hydrophobic and fibrillogenic than Aβ_1–40_ due to the addition of two hydrophobic residues at the N-terminus [64]. Aβ_1–42_ in combination with Cu(II) catalyzes reactive oxygen species production [65]. The high binding affinity of Cu(II) to three histidine residues at positions 6, 13 and 14 facilitates the aggregation of Aβ_1–42_ [66]. The neurotoxicity of Aβ_1–42_ is significantly increased in the presence of Cu(II) compared to Aβ_1-40_, correlating with the capacity to reduce Cu(II) to Cu(I), while forming H_2_O_2_ and oxidative stress [67]. In order to find the concentration level of the Aβ_1–42_-Cu(II) system that produces the highest neurotoxicity, the differentiated SH-SY5Y cells were treated with different concentrations of Aβ_1–42_, Cu(II) and Aβ_1–42_-Cu(II) (molar ratio 1:1). The cell viability of differentiated SH-SY5Y cells after 24 h of treatment was examined by the MTT assay (Figure 3). Figure S1 displays the phase-contrast images. The MTT results confirmed the neurotoxicity of Aβ_1–42_ and Aβ_1–42_ -Cu(II) (molar ratio 1:1). As expected, treatment with Aβ_1–42_ -Cu(II) reduced the cell viability more than Aβ_1–42_ (with the same concentration). This observation emphasized the possible role of Aβ_1–42_-Cu(II) in ROS formation, and this was in agreement with previous studies [67,68]. The 10 µM Aβ_1–42_-Cu(II) (molar ratio 1:1) cell viability was decreased by more than 50% after 24 h of treatment. Therefore, this concentration was chosen as the ROS inducer in the SH-SY5Y in vitro model. A cannabis extract (E2) (see Table S1 for the details of the composition), which contained 81.08% THC, with no detectable CBD, and hemp seed oil (HSO) containing 80% CBD was chosen as the natural source of THC and CBD, which allowed us to compare the effectiveness of THC and CBD to study the oxidative stress induced by the Aβ_1–42_ -Cu(II) system. Irakli et al. [69] recently investigated the nutritional, phytochemical composition and antioxidant properties of hemp seeds. The differentiated neuronal cells were treated with various concentrations of E2 and HSO after pre-treating the SH-SY5Y cells with a 10 µM Aβ_1–42_-Cu(II) complex (molar ratio 1:1).

**Figure 4:**
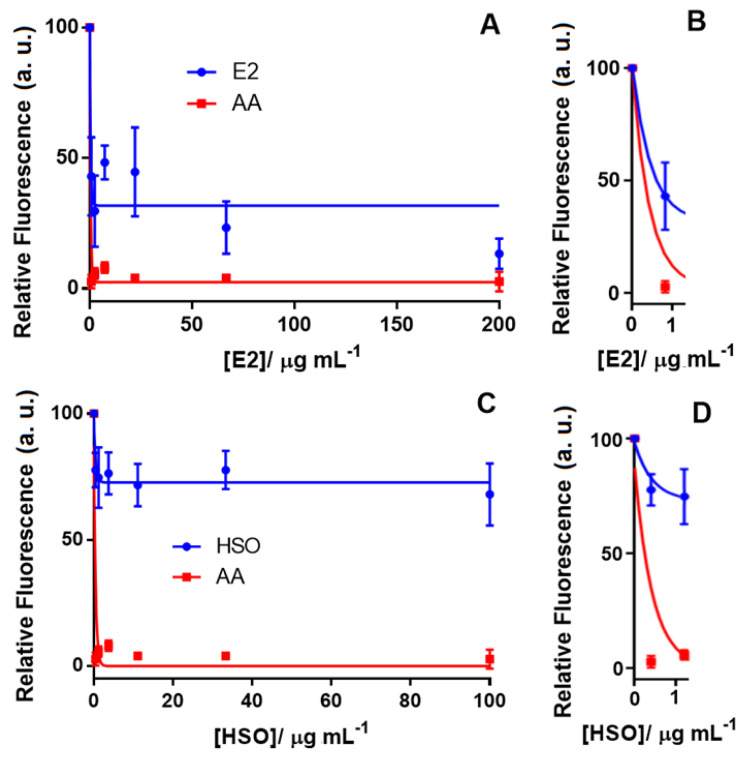
Concentration-response curve for ROS level detection in the differentiated SH-SY5Y cells using the DCFDA assay after treatment with (**A**) the cannabis extract (E2) containing 81% THC (tested concentrations for E2: 0.0, 0.8, 2.5, 7.4, 22.2, 66.7 and 200.00 µg mL^−1^) along with (**B**) the inset. (**C**) hemp seed oil (HSO) containing 80% CBD (tested concentrations for HSO: 0.0, 0.4, 1.2, 3.7, 11.0, 33.0 and 100.0 µg mL^−1^) and (**D**) the inset. In all experiments, the ROS inducer was the Aβ1-42-Cu(II) complex (100% fluorescence intensity), and AA was tested in vehicle only (DMSO) as the positive control. Data show the mean values performed in triplicate (*n* = 3).

Figure 4 shows the neuroprotective effect of E2 and HSO by reducing the ROS level when the Aβ_1–42_-Cu(II) complex was used as the oxidative stress inducer in differentiated SH-SY5Y neuronal cells. In this study, AA was tested as a positive control with a 100% effect on reducing the ROS in differentiated SH-SY5Y neuronal cells. Our results indicated that E2, which contained 81.08% THC and no detectable CBD, reduced the ROS by 60%, while HSO, which contained 80% CBD, was only capable of decreasing the ROS by 25% (Figure 3). E2 displayed an IC_50_ of 0.6 μg mL^−1^ compared to 0.3 μg mL^−1^ of AA, which was used as the positive control. Vitamin E, curcumin and AA are model compounds that have been applied as positive controls to study the antioxidant effects of novel compounds [59]. A chelating agent such as clioquinol was reported to have a nanomolar affinity towards Cu(II) and reduced the formation of H_2_O_2_ by Aβ_1–42_ [43]. Though the mechanism of action for cannabinoids in reducing the oxidative stress created by Aβ_1–42_ is not yet known, we hypothesize that the metal chelating properties of THC and CBD could be the probable explanation. Further investigations on this hypothesis are in progress in our laboratory. Figure S4 shows the concentration-response curve of E2 for ROS level detection in the differentiated SH-SY5Y cells when H_2_O_2_ was used as the ROS inducer. These results reinforced the previous study that reported the effect of THC to inhibit Aβ_1–42_ aggregation [11]. We can speculate that THC had a dual action on the Aβ_1–42_-Cu(II) system by reducing ROS formation and inhibiting Aβ_1–42_ aggregation, which might have prevented neuronal death. However, comparing the antioxidant activity of the phytocannabinoids and cannabis extracts in two AD-like oxidation models suggested a complex antioxidant mechanism that may follow various pathways. Therefore, further studies need to be done to understand the mechanism of the antioxidation and the possible role of cannabinoid receptors that are activated by these compounds. We also performed a series of MTT assays to further investigate the effect of cannabinoids on neuronal viability.

### 2.2. Cell Viability Assay

**Figure 5:**
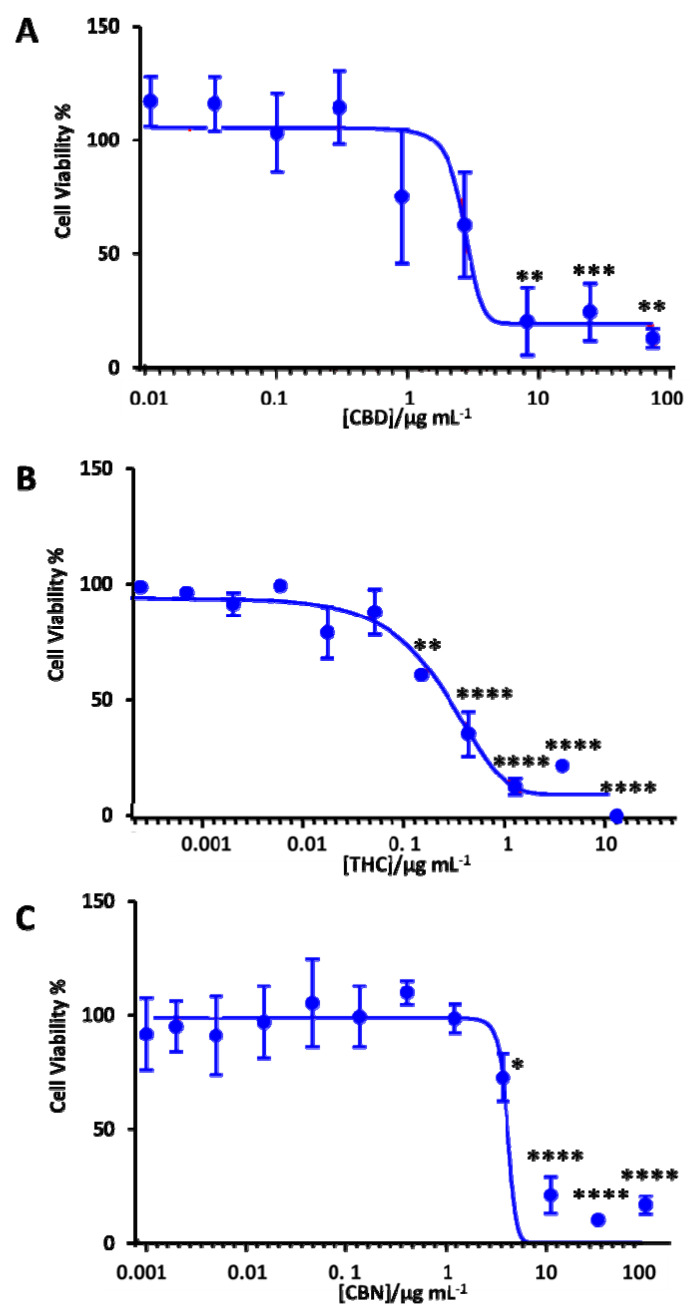
Dose-response curve of the differentiated SH-SY5Y cells exposed to (**A**) CBD, (**B**) THC and (**C**) CBN using the MTT assay after 18 h of treatment with the cannabinoids. Data show the mean values performed in triplicate (*n* = 3). Control (DMSO) vs. cannabinoid shown using the Bonferroni test at * *p* < 0.05, ** *p* < 0.01, *** *p* < 0.001 and **** *p* < 0.0001.

It is critically important to evaluate the toxicity of therapeutic candidates. Therefore, we performed a cell viability assay to determine the neurotoxicity of the cannabis extracts against the differentiated SH-SY5Y human neuronal cells. Table S1 summarizes the chemical profiles of these extracts. In order to examine the impact of CBD, THC and CBN on the neurotoxicity of these extracts, MTT assays were performed. Figure 5 shows the dose-response curve for CBD, THC and CBN against differentiated SH-SY5Y. The dose-response curve for different cannabis extracts is shown in Figure S2. The IC_50_ of these cannabis extracts and cannabinoids (THC, CBD and CBN) was calculated based on the dose-response curve (Table 2). The concentration ranges were from 0.0001 µg mL^−1^ to 13 µg mL^−1^ for THC, from 0.01 µg mL^−1^ to 74 g/mL^−1^ for CBD and from 0.001 µg mL^−1^ to 100 µg mL^−1^ for CBN. While CBD (<1 µg mL^−1^) and CBN (<5 µg mL^−1^) did not show any neurotoxicity, THC treatment reduced the cell viability of differentiated SH-SY5Y by 50% at a 0.6 µg mL^−1^ concentration (Figure 5).

Cannabis extracts E1 and E3 with high THC (>71%) showed a similar effect to pure THC (Table 2). Furthermore, these extracts had a lower IC_50_ in comparison with extracts E7 and E8, which had more than 50% CBD and a low THC proportion. Notably, extracts E7 and E8 (IC_50_ = 11 μg mL^−1^) were less neurotoxic than the pure CBD (IC_50_ = 5 μg mL^−1^). On the other hand, cannabinol (CBN), having a structure like THC (Figure 1), was found to have an IC_50_ of 6.5 μg mL^−1^, which was 10-fold higher than THC. The extracts tested not only had different ratios of THC and CBD, but also other naturally occurring compounds like cannabigerol (CBG) and cannabichromene (CBC) (see Table S1 for the metabolite profile of these cannabis extracts). These other constituents of the extracts tested have their own therapeutic potential, which could either have a synergistic or antagonistic effect in the cells. We are currently in the process of investigating those individual and combined effects of CBG and CBC in our laboratory. Previous studies showed that CBG was non-psychoactive and did not bind to the CB_1_ or CB_2_ receptors, but displayed antioxidant and anti-inflammatory properties [60]. Prior literature indicated that these constituents might synergistically enhance the effect of the primary phytocannabinoids and mitigate their side effects, especially with regards to THC [5]. Recently, di Giacomo et al. [70] reported the neuroprotective and neuromodulatory effects induced by CBD and CBG in rat Hypo-E22 cells and isolated hypothalamus. This information would be useful to find an optimal concentration to attain the beneficial effects of phytocannabinoids in cannabis extracts towards neurodegenerative disease treatment.

To test which proportion (devoid of other cannabinoid constituents) was responsible for these effects, different ratios of pure THC and CBD were investigated as control assays (Figure S3 and Table S2). The ratios tested for cell viability were 90:10, 75:25, 50:50, 25:75 and 10:90 (w/w of CBD:THC). While all the tested THC:CBD solutions were nontoxic below a 1 µg mL^−1^ concentration, the 50:50 ratio of THC:CBD had the highest IC_50_ among all the tested solutions.

**Figure 6:**
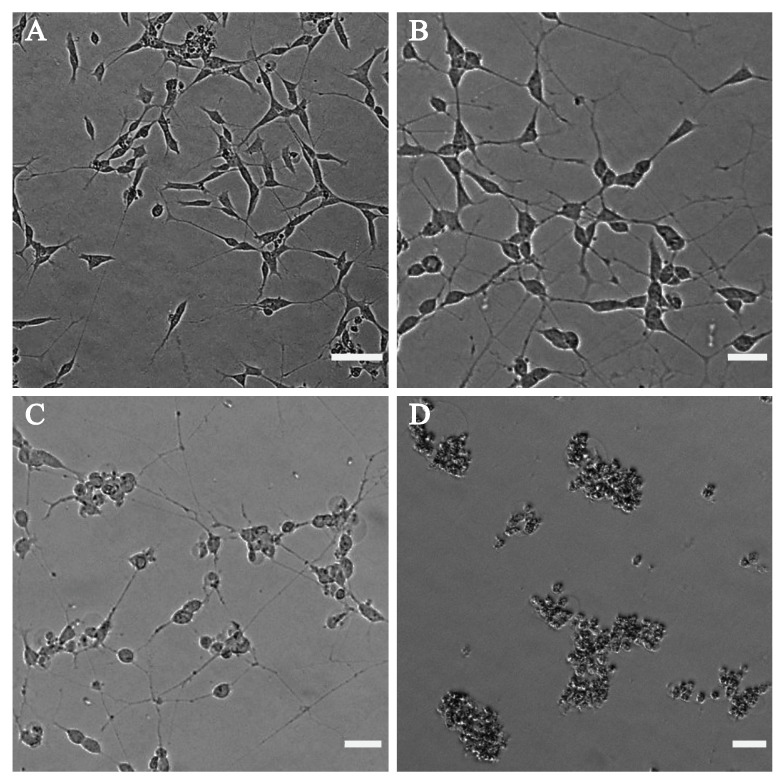
Phase-contrast microscope images of differentiated SH-SY5Y after treatment with (**A**) DMSO as the control, (**B**) 0.1 µg mL^−1^ CBD, (**C**) 2 µg mL^−1^ CBD and (**D**) 10 µg mL^−1^ CBD. CBD at a concentration of 10 µg mL^−1^ (**D**) and above shows dead cells, at 2 µg mL^−1^ (**C**) causes cell body rounding and less affected neurites, whereas at 0.1 µg mL^−1^ (**B**), the phenotype is almost similar to the DMSO ((**A**), vehicle only control) phenotype. Images were taken using the EVOS M5000 Imaging System at 10× magnification. The scalebar indicates 50 µm.

The effect of cannabinoids on the morphology of the differentiated neuronal cells was also investigated by phase-contrast microscopy. Figure 6 shows the phase-contrast microscopy images of the differentiated neuronal SH-SY5Y cells after treatment with CBD. The negative control used for this test was DMSO (Figure 6A), having slender shaped cells with elongated neurites. The neuronal cells displayed negligible bulging but had intact neurites at a concentration of 0.1 μg mL^−1^ of CBD (Figure 6B). Images indicated that cells started to round, and that neurite formation was affected around 2 μg mL^−1^ (Figure 6C), and a concentration of 10 μg mL^−1^ of CBD (Figure 6D) and above caused cell death. Figure S5 shows the phase-contrast images of the differentiated SH-SY5Y cells after treatment with various concentrations of THC.

## 3. Materials and Methods

### 3.1. Materials

Dulbecco’s Modified Eagle’s Medium (DMEM) was obtained from Wisent Bioproducts (Montreal, QC, Canada). Fetal bovine serum (FBS) and 2′,7′-dichlorodihydrofluorescein diacetate (DCFDA) were purchased from Thermo Scientific (Waltham, MA). Δ9-THC (1 mg mL^−1^ in methanol) was acquired from Cayman Chemical Company (Ann Arbour, MI, USA). CBD was provided by Lupos Biotechnology Inc. (Toronto, ON, Canada). 3-(4,5-dimethylthiazol-2-yl)-2,5-diphenyltetrazolium bromide (MTT) reagent, all-trans-retinoic acid, hydrogen peroxide (H_2_O_2_), Cu(II) chloride, dimethyl sulfoxide (DMSO, 99.9%), 1,1,1,3,3,3-hexafluoro-2-propanol (HFIP, 99.0%) and ascorbic acid (AA) were purchased from Sigma-Aldrich (Oakville, ON, Canada). All the chemicals used were of analytical grade and used as received.

### 3.2. Amyloid-β Pretreatment

The Aβ_1–42_ peptide was purchased from Anaspec (Fermont, CA, Canada). Briefly, Aβ_1–42_ peptides were pre-treated by dissolving in HFIP to a final concentration of 1 mg mL^−1^. The resulting suspensions were sonicated for 15 min until the sample solutions became clear. Aβ_1–42_/HFIP solutions were shaken at 400 rpm for 2 h at 4 ± 1 °C. The samples were then left in HFIP and sealed overnight. HFIP was then removed by passing a stream of nitrogen gas across the solvent surface, leaving a clear thin film of peptides at the bottom of the sample vial. The thin film of peptides was re-constituted in DMSO and mixed by vortexing, followed by dilution to the appropriate concentrations with 50 mM PBS containing 100 mM NaCl (pH 7.4). Peptide concentrations were determined by measuring the OD at 280 nm (ε_280_ = 1280 M^−1^) using a NanoDrop 2000 (ThermoScientific, Mississauga, ON, Canada).

### 3.3. Cannabis sativa L. Extracts

Cannabis extracts E1, E2, E3, E7 and E8, which were extracted from *Cannabis sativa L.* by the supercritical carbon dioxide extraction technique, were kindly provided by Lupos Biotechnology Inc. (Toronto, ON, Canada) and were used as received. All the extracts were analyzed by GC-MS at Lupos Biotechnology Inc. (Toronto, ON, Canada) (Table S1). Stock solutions of cannabis extracts were prepared in the DMSO and diluted to the desired concentrations before the treatment. CBD and THC standards as isolated compounds and combined at different ratios CBD:THC (90:10, 75:25, 50:50, 25:75 and 10:90 (*w*/*w*)) were also studied for their antioxidant activity.

### 3.4. Cell Culture and Neuronal Differentiation

The human SHSY5Y neuronal cell line (American Type Culture Collection, Manassas, VA, USA) was maintained in DMEM supplemented with 10% FBS. Cultures were incubated at 37 °C in a humidified 5% CO_2_ atmosphere. The SHSY5Y cells were differentiated by all-trans-retinoic acid (RA) using a well-established protocol as reported before [71]. Briefly, after plating the cells at a cell density of 3.5 × 10^4^ cells per cm^2^ to allow cell adhesion for 24 h, neuronal differentiation was induced by treatment of the cells with 10 µM RA at 37 °C for 72 h under serum-free DMEM conditions.

### 3.5. Cell Viability Assay

The cell viability of the differentiated SH-SY5Y cells was monitored after treatment with various concentrations of cannabis extracts or phytocannabinoids. The differentiated SH-SY5Y cells were also treated with various concentrations of Cu(II) and Aβ_1–42_ in the absence or presence of Cu(II) to find the neurotoxicity that mimics the AD-like oxidative stress condition [53,54]. In all experiments, DMSO was used as the vehicle control. The MTT assay was performed to determine the cell viability after 24 h of treatment with the target extracts or compounds. The MTT assay is a colorimetric technique that uses the reduction of a yellow MTT reagent to measure cell viability. Viable cells contained NAD(P)H-dependent oxidoreductase enzymes, which reduced the MTT reagent to formazan, an insoluble crystalline product with a deep purple color. The standard procedure was followed for the MTT assay: Briefly, 0.2 mg mL^−1^ MTT solution in DMEM were added to each well of a 96 well plate, and the plate was incubated for 4 h at 37 °C in a humidified 5% CO_2_ atmosphere. After 4 h, the media were aspirated, and one hundred microlitres of DMSO were added to each well. The absorbance of the wells was measured at 570 nm, which directly correlates to the number of viable cells, using a microplate reader (Synergy H1 multi-mode reader, BioTek Instruments Inc., Winooski, VT, USA). Cell viability was further confirmed by examination of the cellular morphology with phase-contrast microscopy (EVOS M5000 Imaging System, Thermo-Fisher Scientific, Mississauga, ON, Canada). Dying cells showed extensive rounding of the cell body and condensation of nuclei.

### 3.6. Antioxidant Activity Assay

SH-SY5Y cells at a density of 3.5 × 10^4^ per well were seeded into a dark plate with a clear bottom. After 24 h of incubation at 37 °C, the cells were treated with 10 μM RA at 37 °C for 72 h under serum-free conditions to induce differentiation. The cells were then treated with 100 μM of H_2_O_2_ for 24 h to create oxidative stress in the cells, as reported before [50,51]. The differentiated cells were treated with 5 μM of DCFDA for 30 min to get a measure of the baseline oxidative stress of the cells before being treated with the test compounds. A range of concentrations was used to test the efficacy of the extracts and pure compounds in rescuing the cells after inducing oxidative stress by H_2_O_2_. The cells were incubated with the test compounds for 30 min before measuring the fluorescence at excitation/emission of 492 nm/527 nm using a microplate reader (Synergy H1 multi-mode reader, BioTek Instruments Inc., Winooski, VT, USA). AA was applied as a positive control. To investigate the effect of the Aβ_1–42_-Cu(II) complex as the oxidative stress inducer, the differentiated SH-SY5Y cells were treated with 10 μM of the Aβ_1–42_-Cu(II) complex with a molar ratio of 1:1. The DCF fluorescence intensity of the cells treated with H_2_O_2_ or Aβ_1–42_-Cu(II) was considered as 100%, and the relative fluorescence intensity was calculated for all the samples accordingly.

### 3.7. Statistical Analysis

Data are presented as the mean ± SD. The Bonferroni test was used to evaluate statistical discrepancies between the two groups. *p* < 0.05 was used as the criterion for statistical significance.

## 4. Conclusions

In this proof-of-concept study, we investigated the effects of cannabis extracts with different ratios of THC and CBD on combating the oxidative stress in differentiated neuronal cells. To understand the role of phytocannabinoids, specifically CBD and THC, in the antioxidant activity of the cannabis extracts, we also examined the effect of pure THC, CBD, as well as various mixtures of these two phytocannabinoids. The oxidative stress was induced in vitro in differentiated neuronal SH-SY5Y cells using H_2_O_2_. Our results showed that the 75% THC compound and cannabis extract containing 72% THC could reduce ROS formation by approximately 80%. We also mimicked the AD-like oxidative stress conditions by treating the differentiated neuronal SH-SY5Y cells using Aβ_1–42_-Cu(II) complexes, which also triggered ROS formation. Our results clearly showed the significant impact of cannabis extracts with high THC to combat ROS formation induced by the Aβ_1–42_-Cu(II) in vitro model. However, CBD was not observed to exhibit as high an antioxidant activity as THC under these experimental conditions. The antioxidant activity of cannabis extracts that had a lower percentage of THC suggested that other natural compounds in these extracts might also have had antioxidant activity or a synergetic effect. Our results provide fundamental information on the antioxidant activity of cannabinoids on neuronal cells towards developing a novel therapeutic approach for oxidative stress therapy. Further studies are required to understand the role of cannabinoid receptors, as well as other receptors that may be activated by these compounds in SH-SY5Y cells. Research efforts towards understanding the molecular mechanisms underlaying the antioxidant activities of phytocannabinoids is in progress in our laboratory.

## Figures and Tables

**Table 1:** ROS detection assay in differentiated SH-SY5Y cells using H_2_O_2_ as the oxidative stress inducer.

Test Compound	CBD%	THC%	IC_50_ (μg mL^−1^) *
Ascorbic Acid	---	---	0.25
THC	---	98	0.4_4_
10:90	10	90	2.5_4_
25:75	25	75	0.4_4_
50:50	50	59	0.5_4_
75:25	75	25	1_4_
90:10	90	10	5_4_
CBD	98	---	42.7_1_
E3	N.D.^#^	71.08	0.4_4_
E8	50.34	3.9	0.5_4_
E2	N.D.^#^	81.1	0.7
E7	64.34	11.54	0.6_4_
E1	N.D.^#^	72.88	1.2_4_

* Results are expressed as the mean of triplicates. Average values followed by different numbers (subscript numbers:1–4) differ by the Bonferroni test at *p* < 0.05. ^#^ Not detected.

**Table 2:** IC_50_ of THC, CBD, cannabinol (CBN) and cannabis extracts (E) against differentiated SH-SY5Y cells as determined by the MTT assays.

Test Compounds	CBD%	THC%	IC_50_ (μg mL^−1^) *
THC	---	98	0.6
E3	N.D.^#^	71.80	0.6
E1	N.D.^#^	72.88	0.7
CBD	98	---	5
CBN	---	---	6.5
E2	---	81.10	7
E7	64.34	11.54	11
E8	50.34	3.90	11

* Results are expressed as the mean of triplicates. Average values followed by different numbers (1–4) differ by the Bonferroni test at *p* < 0.05. ^#^ Not detected.

## Supplementary Materials

The following are available online at https://www.mdpi.com/1424-8247/13/11/328/s1, Figure S1: Phase-contrast microscope images of (**A**) the differentiated SH-SY5Y without treatment; (**B**) after treatment with 10 μM Aβ1–42; (**C**) 10 μM Cu(II); and (**D**) 10 μM Aβ-Cu2+ (molar ratio 1:1) complex for 24 h. Scale bars indicate 400 μm, Figure S2: Dose-response curve of MTT cell viability assay with different cannabis extracts (**A**) E1, (**B**) E3, (**C**) E7, (**D**) E8, (**E**) E2 in the differentiated SH-SY5Y cells, Figure S3: Dose-response curve of MTT cell viability assay in the differentiated SH-SY5Y cells with CBD:THC ratios (**A**) 90:10, (**B**) 75:25, (**C**) 50:50, (**D**) 25:75, and (**E**) 10:90, Figure S4: Concentration-response curve for monitoring the ROS level in differentiated SH-SY5Y cells using DCFDA assay after treatment (**A**) with cannabis extract E2 and (**B**) inset displaying the data from low concentrations of E2, Figure S5: Phase-contrast microscope images of differentiated SH-SY5Y after treatment with (**A**) DMSO as the control (vehicle only), (**B**) 0.1 µg mL^−1^ THC, (**C**) 2 µg.mL^−1^ THC, and (**D**) 10 µg mL^−1^ THC, Table S1: Chemical profile of cannabis extracts obtained from GC-MS analysis, Table S2: IC50 of CBD:THC solutions obtained from MTT cell viability assay.
