# Supplementary material for: Attenuation of Oxidative Stress by Cannabinoids and Cannabis Extracts in Differentiated Neuronal Cells
Source: Pharmaceuticals (Basel). 2020 Oct 22;13(11):328. doi: 10.3390/ph13110328 (PMC7690570; doi:10.3390/ph13110328)
Supplement: Supplementary file 1 [file pharmaceuticals-13-00328-s001.zip › pharmaceuticals-923892-supplementary.docx]

**Supporting Information**

Attenuation of Oxidative Stress by Cannabinoids and Cannabis Extracts in Differentiated Neuronal Cells

Aruna Raja ^1,†^, Soha Ahmadi ^1,2†^, Fernanda de Costa ^3^, Nan Li ^3^ and Kagan Kerman ^1,^*

^1^ Department of Physical and Environmental Sciences, University of Toronto Scarborough, 1265 Military Trail, Toronto, M1C 1A4 ON, Canada; aruna.raja@utoronto.ca (A.R.); soha.ahmadi@mail.utoronto.ca (S.A.)

^2^ Department of Chemistry, University of Toronto, 80 St. George Street, Toronto,M5S 3H6 ON, Canada

^3^ Lupos Biotechnology Inc., 221 Morrish Road, Toronto, M1C 1E9 ON, Canada;
fernanda.decosta@lupos.ca (F.D.); nan.li@lupos.ca (N.L.)

***** Correspondence: kagan.kerman@utoronto.ca

† These authors contributed equally to this work.


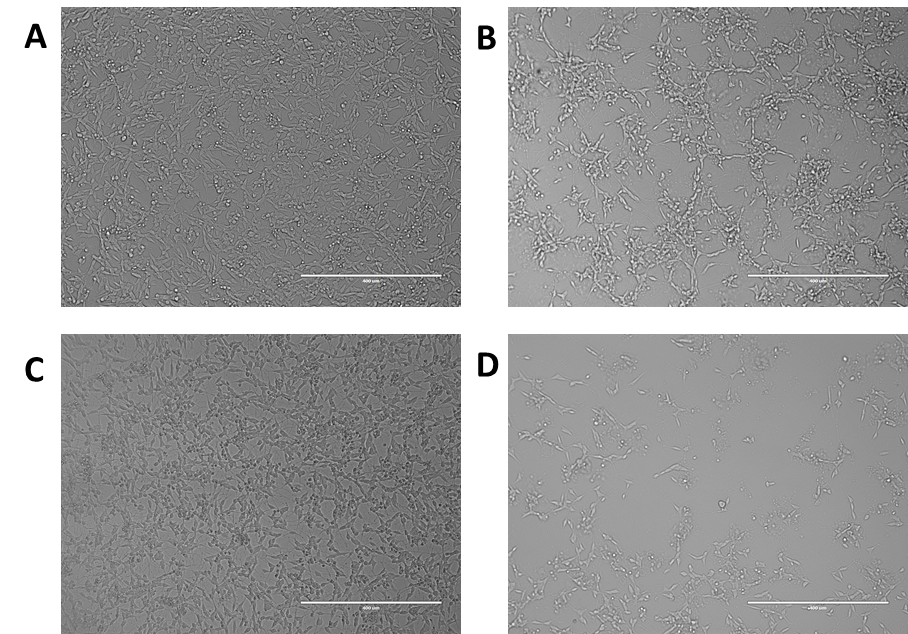


**Figure 1.** Phase-contrast microscope images of (A) the differentiated SH-SY5Y without treatment; (B) after treatment with 10 μM Aβ_1-42_; (C) 10 μM Cu(II); and (D) 10 μM Aβ-Cu^2+^ (molar ratio 1:1) complex for 24 h. Scale bars indicate 400 μm.


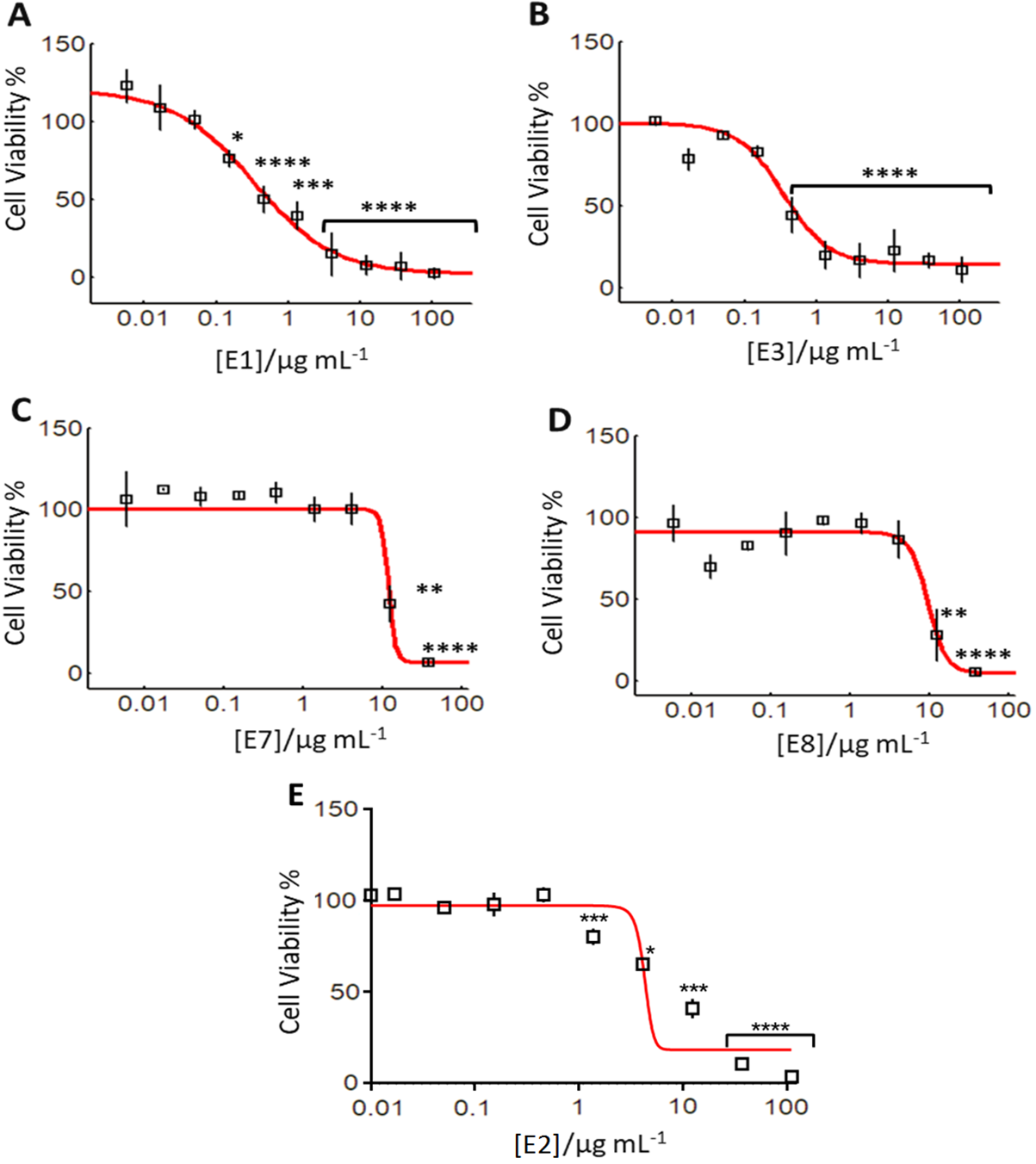


**Figure 2.** Dose-response curve of MTT cell viability assay with different cannabis extracts (A) E1, (B) E3, (C) E7, (D) E8, (E) E2 in the differentiated SH-SY5Y cells. Values are expressed as mean of experiments performed in triplicate (*n* = 3). Control (vehicle only, DMSO) vs. response (extracts) data are shown using Bonferroni test at ** *p* < 0.01, *** *p* < 0.001 and **** *p* < 0.0001.


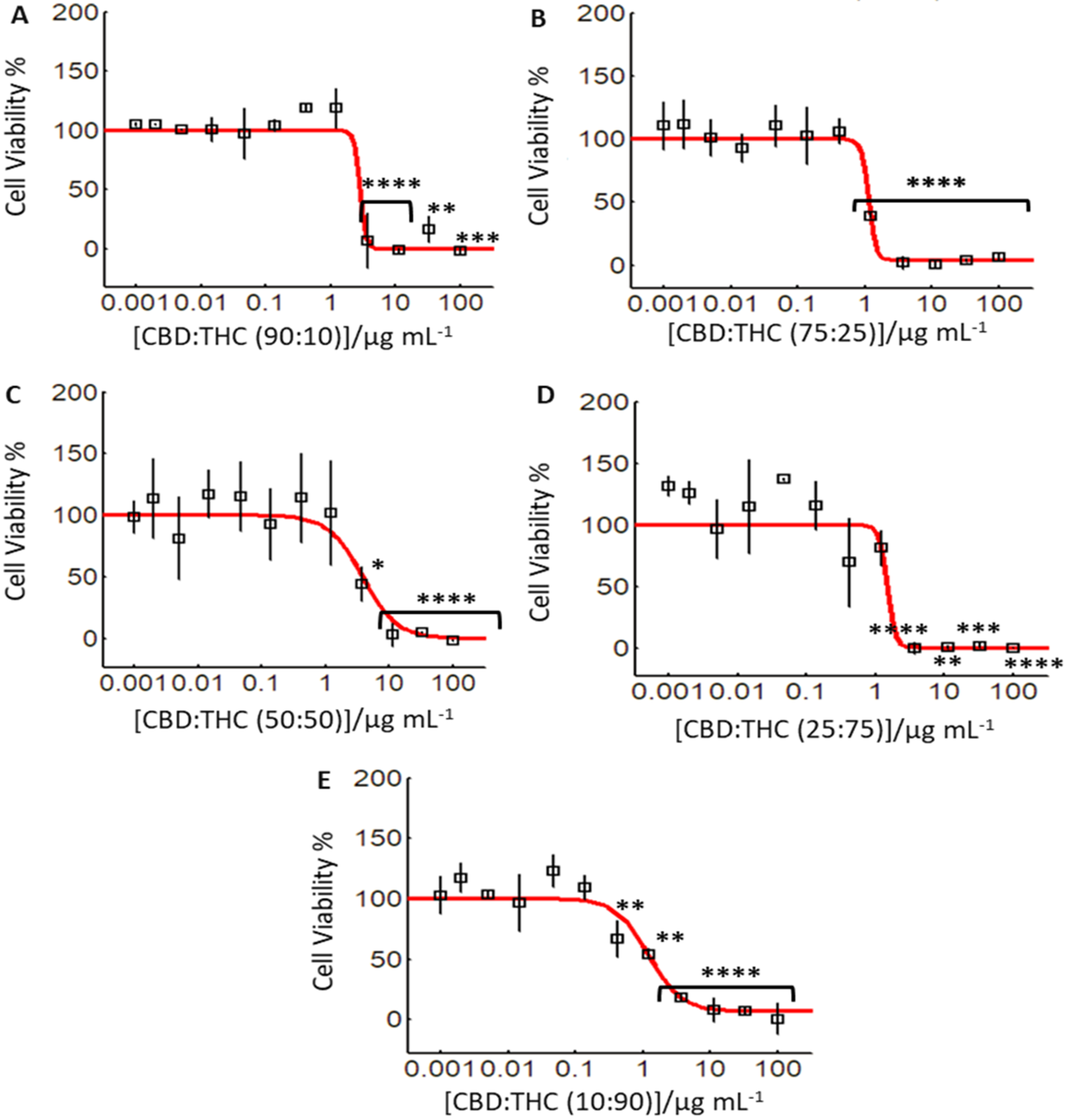


**Figure S3.** Dose-response curve of MTT cell viability assay in the differentiated SH-SY5Y cells with CBD:THC ratios (A) 90:10, (B) 75:25, (C) 50:50, (D) 25:75, and (E) 10:90. Values are expressed as mean of experiments performed in triplicate (*n* = 3). Control (vehicle only, DMSO) vs. response data are shown using Bonferroni test at * *p* < 0.05, ** *p* <0.01, *** *p* < 0.001 and **** *p* <0.0001.


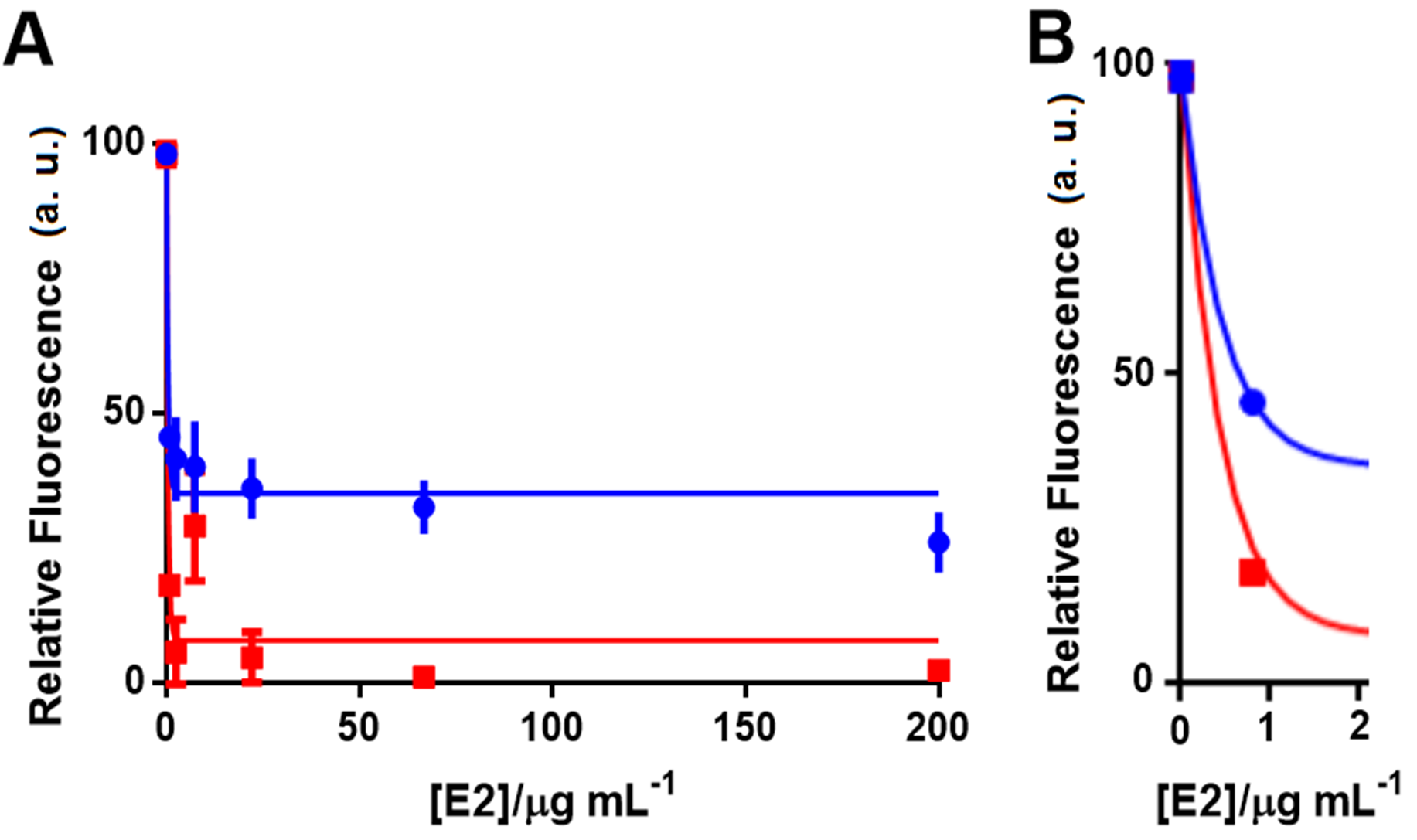


**Figure S4.** Concentration-response curve for monitoring the ROS level in differentiated SH-SY5Y cells using DCFDA assay after treatment (A) with cannabis extract E2 and (B) inset displaying the data from low concentrations of E2. In all experiments, H_2_O_2_ was used as the ROS inducer (100% fluorescence intensity) and ascorbic acid (AA) was tested in vehicle only (DMSO) as positive control. Data show the average of mean values determined in triplicate measurements (*n* = 3). Control (DMSO) vs. E2 data are shown using Bonferroni test at *p* < 0.0001.

| 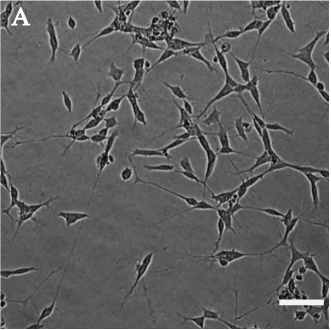 | 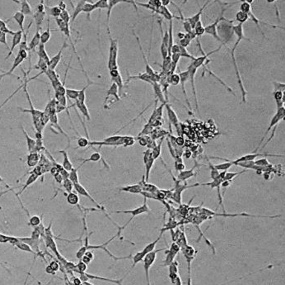 |
| --- | --- |
| 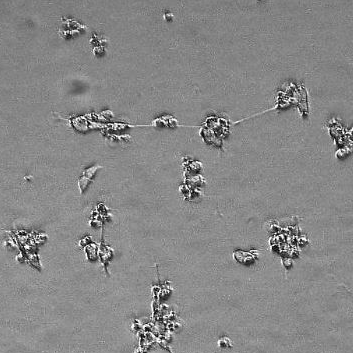 | 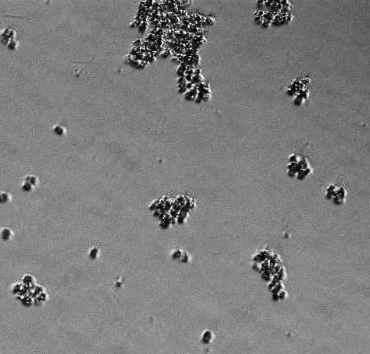 |

**Figure S5.** Phase-contrast microscope images of differentiated SH-SY5Y after treatment with (A) DMSO as the control (vehicle only), (B) 0.1 µg mL^-1^ THC, (C) 2 µg mL^-1^ THC, and (D) 10 µg mL^-1^ THC. THC at the concentration of 10 µg mL^-1^ and above shows dead cells, at 2 µg mL^-1^ causes cell body rounding up and at 0.1 µg mL^-1^ the phenotype is almost similar to DMSO (vehicle only, control) phenotype. Phase-contrast microscopy images were taken using EVOS M5000 Imaging System at 10X magnification. The scalebar indicates 50 µm.

**Table S1**. Chemical profile of cannabis extracts obtained from GC-MS analyses.

| **Extracts** | **CBD%** | **THC%** | **CBC%** | **CBG%** | **CBN%** |
| --- | --- | --- | --- | --- | --- |
| E1 | N.D. | 72.9 | 1.2 | 7.7 | 3.9 |
| E2 | N.D. | 81.1 | 0.7 | 3.1 | 0.7 |
| E3 | N.D. | 71.8 | 2.12 | 4.5 | 0.9 |
| E7 | 64.3 | 11.5 | N.D. | 2.9 | 0.4 |
| E8 | 50.3 | 3.9 | N.D. | 1.4 | 0.1 |

CBD: Cannabidiol, THC: Δ⁹-tetrahydrocannabinol, CBC: Cannabichromene, CBG: Cannabigerol, CBN: Cannabinol, N.D.: Not detected

**Table 2.** IC_50_ of CBD:THC solutions obtained from MTT cell viability assay. .

| **CBD: THC ratio** | **IC_50_, μg mL^−1^** |
| --- | --- |
| 10:90 | 1 |
| 25:75 | 2.5 |
| 50:50 | 6 |
| 75:25 | 2 |
| 90:10 | 5 |

CBD: Cannabidiol, THC: Δ⁹-tetrahydrocannabinol
